# LncRNA BANCR facilitates vascular smooth muscle cell proliferation and migration through JNK pathway

**DOI:** 10.18632/oncotarget.21603

**Published:** 2017-10-07

**Authors:** He Li, Xian Liu, Lan Zhang, Xueqi Li

**Affiliations:** ^1^ Department of Cardiology, The Fourth Hospital of Harbin Medical University, Harbin, China

**Keywords:** atherosclerosis, vascular smooth muscle cell, lncRNAs, BANCR, JNK

## Abstract

Deregulated migration and proliferation of vascular smooth muscle cells (VSMCs) acts a crucial role in the pathogenesis of many cardiovascular diseases such as atherosclerosis, coronary heart disease and hypertension. Long noncoding RNAs (lncRNAs) play crucial functional roles in a lot of biological processes such as cell development, cell proliferation, differentiation and invasion. In our study, we demonstrated that the BANCR expression level was upregulated in the atherosclerotic plaques tissues compared to in the normal vessels tissues. TNF-α could emhance the VSMCs proliferation. The expression level of BANCR and p-JNK were upregulated and activated in the proliferating VSMCs. Overexpression of BANCR enhanced VSMCs proliferation and migration. Elevated expression of BANCR induced JNK activation, which can be decreased by the specific JNK inhibitor SP600125. We demonstrated that ectopic expression of BANCR increased the VSMCs proliferation and migration through activating JNK pathway. These data suggested that lncRNA BANCR acts a crucial role in the regulating VSMCs proliferation and migration partly by activating the JNK pathway.

## INTRODUCTION

Vascular smooth muscle cells (VSMCs) are the major construction of the vasculature and act crucial roles in maintaining blood pressure and vessel tone [1–5]. Deregulated proliferation of VSMCs is a central factor in various cardiovascular diseases including hypertension, coronary heart disease and atherosclerosis [2, 6–9]. VSMCs proliferation and migration can be stimulated by a lot of cytokines and growth factors such as TNF-α and leptin, which act an important role in the development of these diseases [10–12]. However, the mechanism of how these moleculars modulate the VSMCs proliferation remains unclear.

Long noncoding RNAs (lncRNAs) are defined as RNAs with length more than 200 nucleotides with limited or without palpable protein-coding functions [13–18]. Recently, various studies have demonstrated that lncRNAs play important functional roles in biological processes such as cell proliferation, metastasis, development, differentiation, migration and invasion [19–24]. Notably, various lncRNAs were found to be deregulated in a lot of tumors including gastric cancer, colon cancer, hepatocellular carcinoma, gallbladder carcinoma and breast cancer [13–15, 25, 26]. BRAF-activated noncoding RNA (BANCR) is a 693-bp lncRNA firstly identified in melanoma cells [27, 28]. Deregulated expression of BANCR was found in lung cancer, colorectal cancer, gastric cancer and bladder cancer [29–32]. BANCR modulated cell migration, invasion and proliferation and acted as a potential tumor suppressor or oncogene [28, 29, 33, 34]. However, its expression, function and roles in VSMCs are unknown and need to be investigated.

In this study, we showed that the expression of BANCR was upregulated in the atherosclerotic plaques tissues compared to that in normal vessels tissues. The expression of BANCR and p-JNK were upregulated and activated in the proliferating VSMCs. Elevated expression of BANCR promoted VSMCs proliferation and migration.

## RESULTS

### BANCR expression was upregulated in atherosclerotic plaques tissues

**Figure 1:**
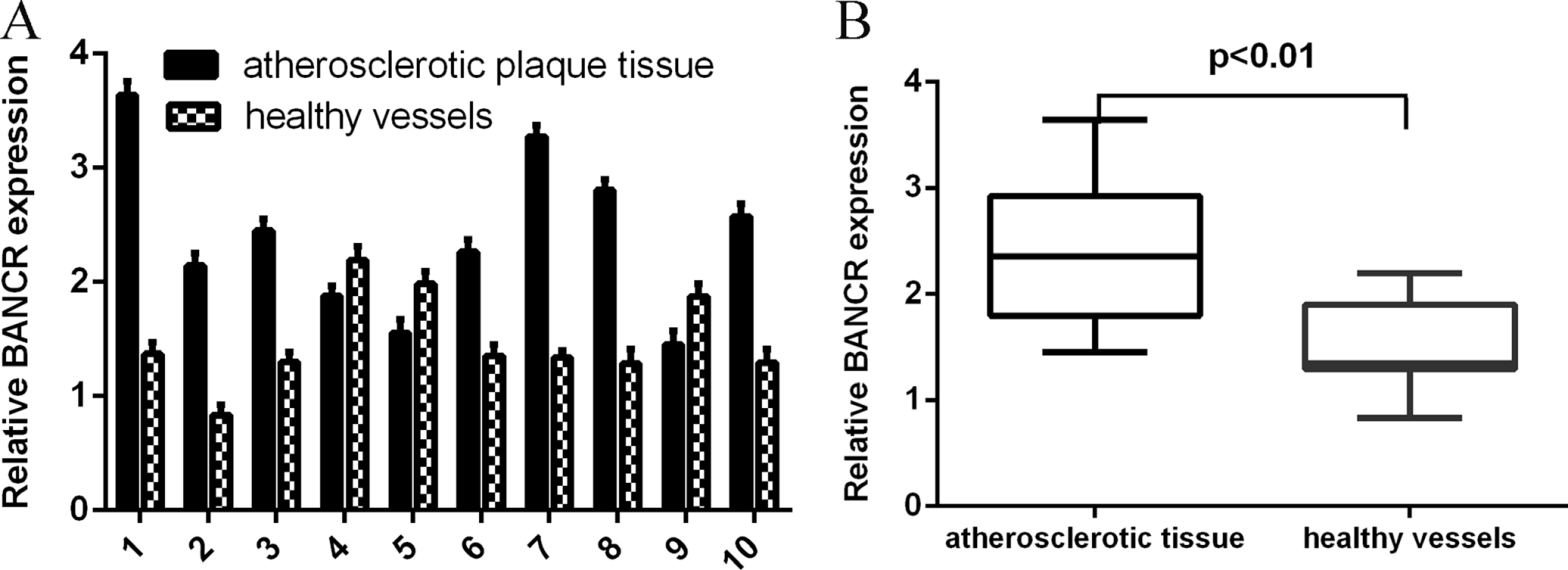
BANCR expression was upregulated in atherosclerotic plaques tissues (**A**) The expression of BANCR in normal vessels tissues and human atherosclerotic plaques tissues was determined by qRT-PCR. (**B**) The expression level of BANCR was upregulated in atherosclerotic plaques tissues compared to in the normal vessels tissues. ^***^p < 0.001.

We first measured the BANCR expression in the atherosclerosis tissues. The expression of BANCR in normal vessels tissues and human atherosclerotic plaques tissues was shown in the Figure 1A. The expression level of BANCR was upregulated in atherosclerotic plaques tissues compared to in the normal vessels tissues (Figure 1B).

### BANCR expression was upregulated by TNF-α in the VSMCs

**Figure 2:**
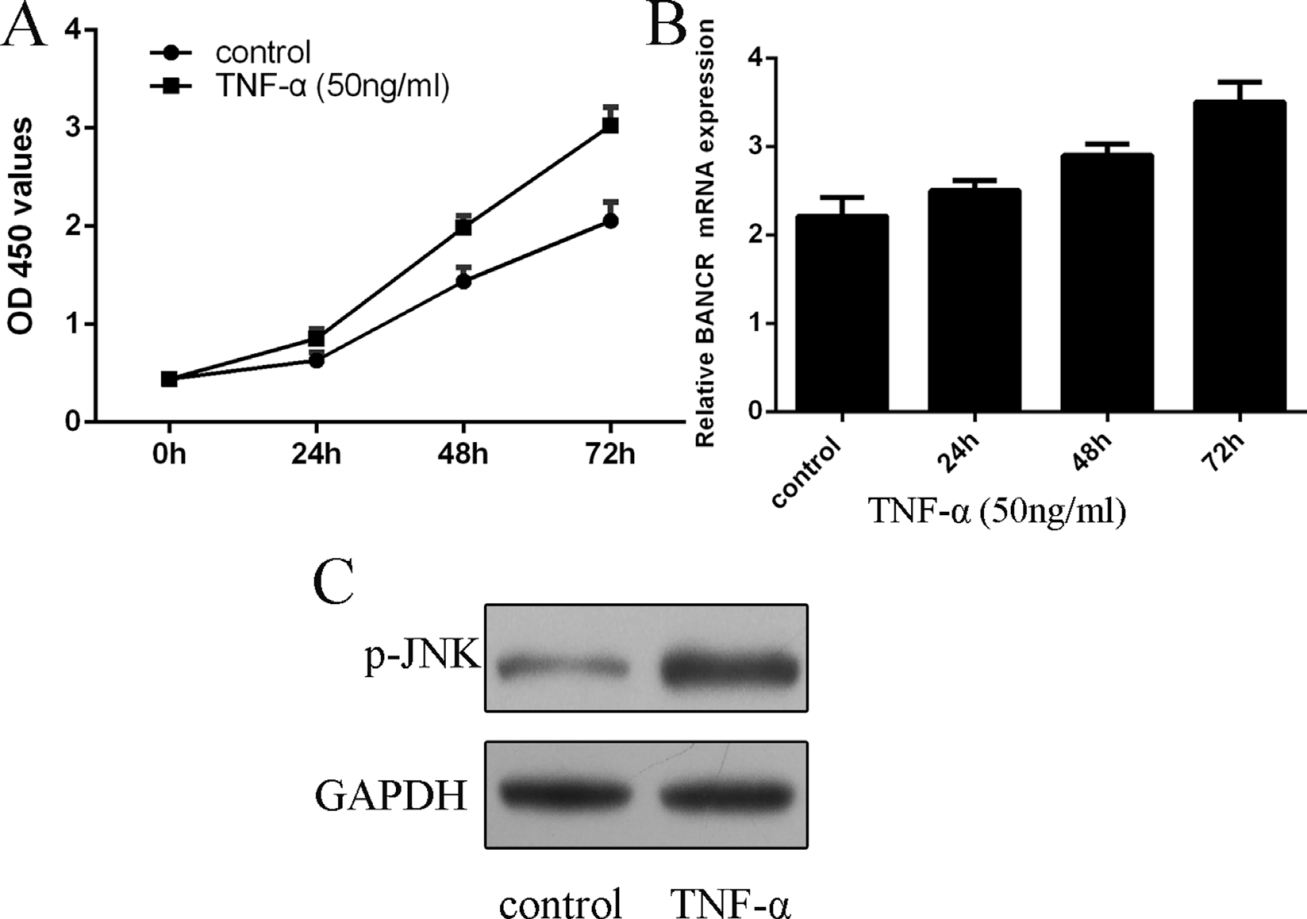
BANCR expression was upregulated by TNF-α in the VSMCs (**A**) TNF-α (50 ng/ml) promoted VSMCs proliferation. (**B**) The expression of BANCR in the proliferating VSMCs was measured by qRT-PCR. (**C**) The expression of p-JNK was detected by western blot. ^*^p < 0.05, ^**^p < 0.01 and ^***^p < 0.001.

We further measured the expression of BANCR in the proliferating VSMCs. We confirmed that TNF-α (50 ng/ml) promoted VSMCs proliferation (Figure 2A). As shown in the Figure 2B, the expression of BANCR was upregulated in the proliferating VSMCs (Figure 2B). In addition, the expression of p-JNK was activated in the proliferating VSMCs (Figure 2C).

### BANCR promoted the VSMCs proliferation and migration

**Figure 3:**
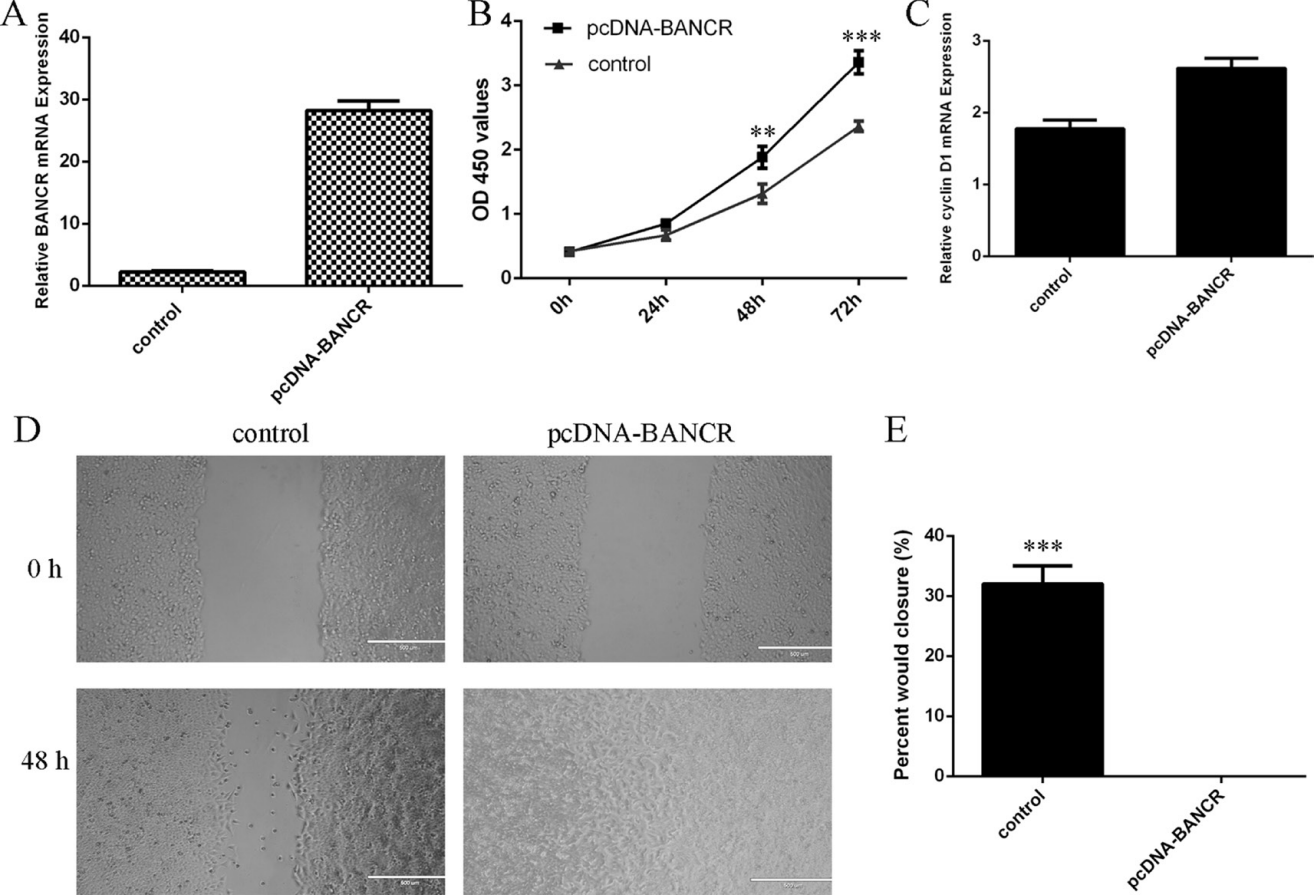
BANCR promoted the VSMCs proliferation and migration (**A**) The expression of BANCR in the VSMCs after treated with pcDNA- BANCR was measured by qRT-PCR. (**B**) Elevated expression of BANCR promoted VSMCs proliferation. (**C**) Overexpression of BANCR increased cyclin D1 expression in the VSMCs. (**D**) Ectopic expression of BANCR promoted VSMCs migration. (**E**) The relative migration wound was shown. ^**^p < 0.01 and ^***^p < 0.001.

To measure the functional role of BANCR in the VSMCs, we treated the VSMCs with pcDNA-BANCR. The expression of BANCR was significantly upregulated in the VSMCs after treated with pcDNA- BANCR (Figure 3A). Elevated expression of BANCR promoted VSMCs proliferation (Figure 3B). We also demonstrated that overexpression of BANCR increased cyclin D1 expression in the VSMCs (Figure 3C). Ectopic expression of BANCR promoted VSMCs migration (Figure 3D and 3E).

### BANCR induced JNK signal pathway activation

**Figure 4:**
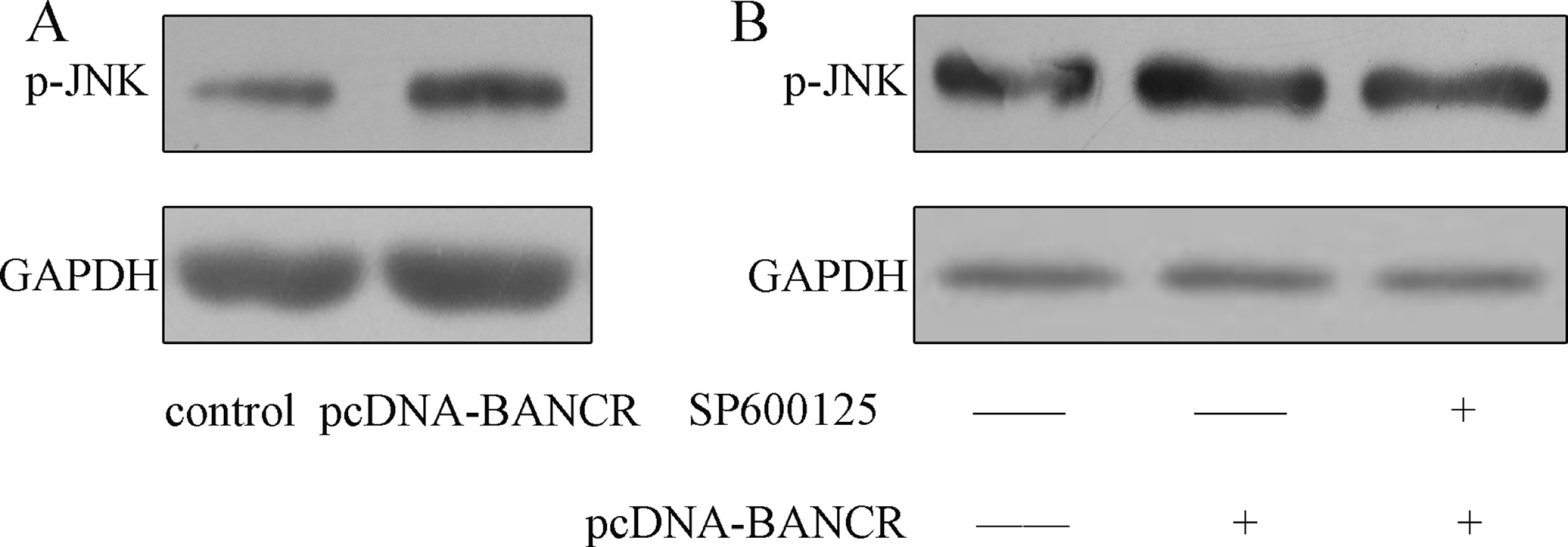
BANCR induced JNK signal pathway activation (**A**) The expression of p-JNK was detected by western blot. BANCR induced JNK signal pathway activation. (**B**) The expression of p-JNK in the VSMSs was detected by western blot.

As shown in the Figure 4A, the JNK was activated in the pcDNA-BANCR transfected VSMCs compared with control transfected cells. Moreover, this effect was inhibited by treated with SP600125, which was the inhibitor of the JNK (Figure 4B).

### BANCR promoted VSMCs proliferation and migration via regulating JNK

**Figure 5:**
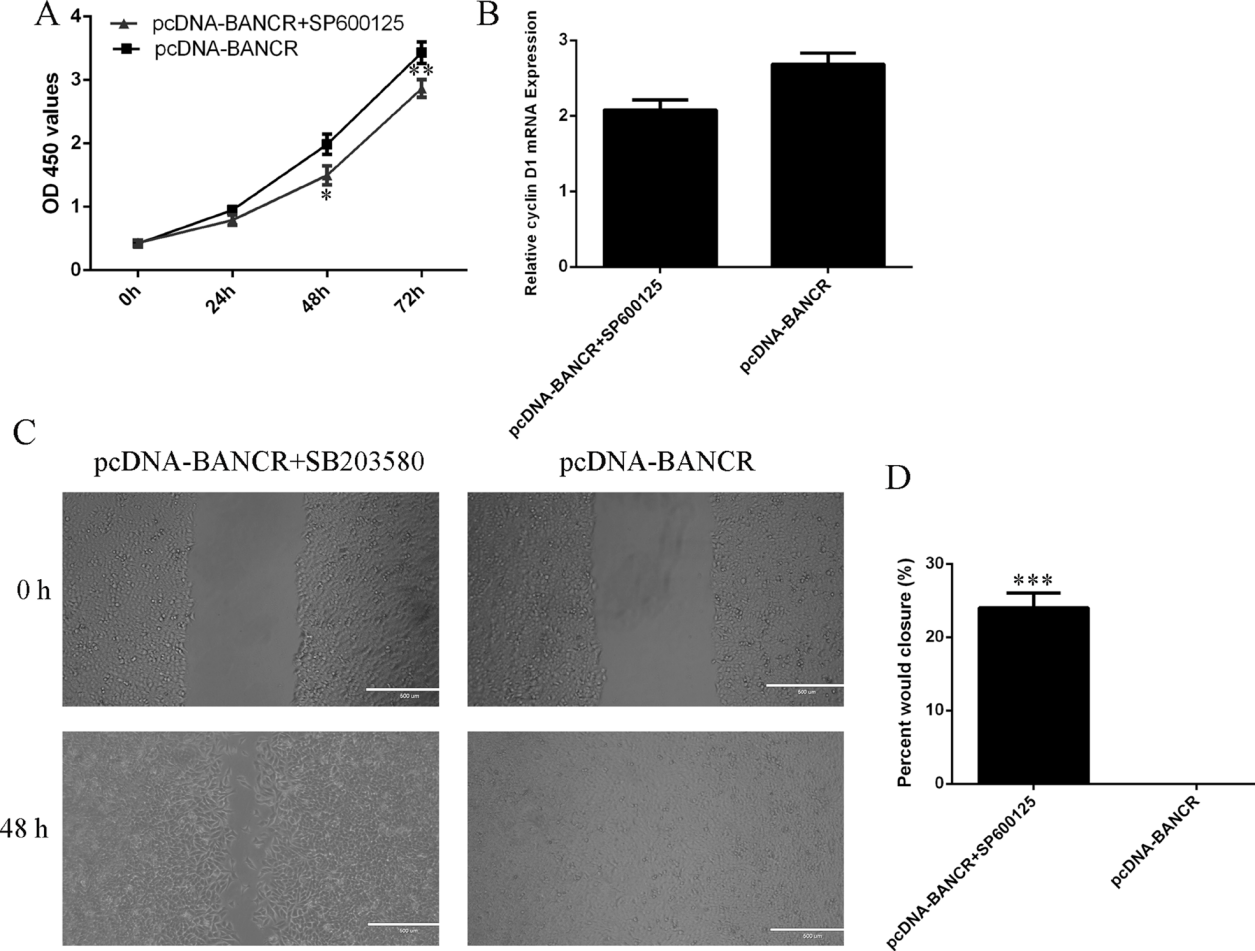
BANCR promoted VSMCs proliferation and migration via regulating JNK (**A**) The VSMCs proliferation was significantly decreased after treated with specific JNK inhibitor SP600125. (**B**) The cyclin D1 expression was measured by qRT-PCR. (**C**) The BANCR-induced VSMCs migration was also decreased after treated with specific JNK inhibitor SP600125. (**D**) ^*^p < 0.05, ^**^p < 0.01 and ^***^p < 0.001.

In order to detect the effect of JNK on the BANCR-regulated cell migration and proliferation, the VSMCs that were treated with specific JNK inhibitor SP600125. The VSMCs proliferation was significantly decreased after treated with specific JNK inhibitor SP600125 (Figure 5A). In line with this, cyclin D1 expression was also suppressed in the VSMCs (Figure 5B). The BANCR-induced VSMCs migration was also decreased after treated with specific JNK inhibitor SP600125 (Figure 5C, 5D).

## DISCUSSION

In this study, we showed that the expression level of BANCR was upregulated in the atherosclerotic plaques tissues compared to in the normal vessels tissues. We demonstrated that TNF-α promoted the VSMCs proliferation. The expression level of BANCR was upregulated and the expression of p-JNK was activated in the proliferating VSMCs. Elevated expression of BANCR enhanced the VSMCs proliferation and migration. Overexpression of BANCR can induce the JNK activation, which could be suppressed by the specific JNK inhibitor SP600125. We also demonstrated that ectopic expression of BANCR promoted VSMCs proliferation and migration by regulating JNK. These data suggested that lncRNA BANCR played an important role in regulating VSMCs proliferation and migration partly through activating the JNK pathway.

Previous evidences demonstrated that lncRNAs played a critical role in cardiovascular biology and disease [35–37]. LncRNAs can regulate the vessel function and growth and control phenotype of the smooth muscle cells [38–40]. For example, Wu et al. [41]. demonstrated that lincRNA-p21 regulated the VSMCs apoptosis and proliferation during atherosclerosis. They also showed that lincRNA-p21expression was downregulated in these patients with coronary artery disease. Zhao et al. [42]. showed that lncRNA HIF1a-AS1 was upregulated in this thoracoabdominal aorta aneurysm tissues and HIF1a-AS1 regulated the VSMCs proliferation and apoptosis. BANCR is a 693-bp lncRNA original identified in melanoma cells [27]. Increasing studies suggested that BANCR played an important role in the development of tumors [28, 31, 32]. For example, Li et al. [31]. showed that BANCR expression was upregulated in gastric cancer tissues and the expression of BANCR was correlated with the lymph node metastasis, tumor depth, clinical stage and distant metastasis. Zhang et al. [34]. demonstrated that overexpression of BANCR promoted the gastric cancer cell proliferation and suppressed the cell apoptosis through regulating the NF-κB1 expression. Li et al. [28]. showed that BANCR expression was upregulated in the malignant melanoma tissues and cell lines. Elevated expression of BANCR promoted the melanoma cell proliferation by activating ERK1/2 and JNK pathway. However, its expression and function roles in VSMCs are still unknown and need to be investigated. In our study, we firstly measured the BANCR expression in the atherosclerosis tissues. We demonstrated that the expression level of BANCR was upregulated in the atherosclerotic plaques tissues compared to in the normal vessels tissues. We also showed that TNF-αpromoted VSMCs proliferation. The expression of BANCR was upregulated and the expression of p-JNK was activated in the proliferating VSMCs. Elevated expression of BANCR promoted the VSMCs proliferation and migration. These result suggested that BANCR played an important role in regulating VSMCs proliferation and migration.

MAPK pathway families act crucial roles in a large number of cell programs such as cell proliferation, differentiation, apoptosis, migration, and invasion [43–45]. Activation of Erk, JNK and p38 MAPK pathways can induce tumor cell development, differentiation, proliferation and other cell processes [46–48]. Previous studies also showed that JNK acted a critical role in VSMCs proliferation and migration [49–51]. Nagayama et al. [52]. demonstrated that Exendin-4 suppressed VSMCs migration and proliferation by Angiotensin II through inhibiting JNK and ERK1/2 signaling pathways. Zhang et al. [53]. also showed that overexpression of miR-92a suppressed the H2O2-induced VSMC apoptosis through directly regulating the mitogen-activated protein kinase kinase 4 (MKK4) and JNK pathway. Recently, Jiang et al. [32]. showed that BANCR increased lung carcinoma migration and proliferation through MAPK pathways. Li et al. [28]. also demonstrated that BANCR promoted malignant melanoma cell proliferation through activating MAPK pathway. In line with this, we showed that overexpression of BANCR induced the JNK activation, which could be suppressed by the specific JNK inhibitor SP600125. We also demonstrated that ectopic expression of BANCR promoted VSMCs proliferation and migration by regulating JNK.

In conclusion, we demonstrated that the expression of BANCR was upregulated in the atherosclerotic plaques tissues compared to in the normal vessels tissues. The expression of BANCR was upregulated in the proliferating VSMCs. Elevated expression of BANCR promoted the VSMCs proliferation and migration through activating JNK pathway. These results suggested that lncRNA BANCR played an important role in regulating VSMCs proliferation and migration partly through activating the JNK pathway.

## MATERIALS AND METHODS

### Cell culture and tissue samples

Human atherosclerosis plaques samples in coronary artery patients and healthy vessels tissues were collected from the patients undergoing the surgery in our Departement. This study was approved by the Research Ethics Committee of the Fourth Hospital of Harbin Medical University. Written informed consent was obtained by all patients and this study aslo was complied with the Declaration of Helsinki. The VSMCs were purchased from Cascade Biologics (Portland) and kelpt in the recommended culture conditions. The pcDNA-BANCR and pcDNA-control was purchased from Genama (Shanghai, China) and transfected to the VSMCs by using the DharmaFECT1 Reagent (Dharmacon, TX, USA) according to the information.

### Real-time RT-PCR

Total RNAs were isolated from cells by using TRIzol kit (Invitrogen, USA) following to the manufacturer’s information. The qRT-PCR reaction was measured with the SYBR Green mix (Bio-Rad, CA, USA). The expression of GAPDH was monitored as the internal control. The real-time PCR was performed on the iQ-5 system (Bio-Rad) and the average Ct was used for calculations. The bellow primer was used: BANCR, Forward: 5′-ACAGGACTCCATGGCAAACG-3′; reverse: 5′-ATGAAGAAAGCCTGGTGCAGT-3′. GAPDH, Forward: 5′-GGGAGCCAAAAGGGTCAT-3′; reverse: 5′-GAGTCCTTCCACGATACCAA-3′.

### Western blotting

Total cellular protein was isolated by using lysis buffer and the protein concentration was measured by BCA assay (Pierce, Rockford, IL, USA). Equal proteins were separated on the 10% SDS and transferred to the PVDF membrane. After blocking with 5% non-fat milk, the membrane was incubated with the primary antibodies (p-JNK and GAPDH, Sigma) and then incubated with horseradish peroxidase-conjugated secondary antibodies (Santa Cruz, CA). The protein signal was visualized by using the ECL chemiluminescence (Pierce).

### Cell proliferation and invasion

For cell proliferation assay, the cell was seeded in the 96-well plate and cultured for 0, 24, 48, 72 hours. Cells were incubated with the MTT (3-(4, 5-dimethylthiazol-2-yl)-2,5-diphenyltetrazolium bromide) for 1 hour. The absorbance OD was determined using the plate reader (PerkinElmer, MA, USA). For the invasion analysis, the insert was incubated with Matrigel (Sigma, St Louis, USA). Cell was cultured on the upper chamber with no-serum medium. 10% serum was added to the lower chamber and the cell was cultured for 48 hours. The cell which invaded into the lower surface was fixed with the paraformaldehyde and stained with crystal.

### Statistical analysis

The result was expressed as the mean ± standard deviation (SD). Statistical assay was done using the SPSS program ver. 17 (SPSS, Chicago, USA). The signicant difference between groups was determined using Student’s *t*-test or ANOVA. *P* < 0.05 was thought to be statistically significant.
